# Evaluation of Radiological and Functional Outcome of Intra-articular Proximal Tibia Plateau Fracture Treated with Plating

**DOI:** 10.5704/MOJ.2303.011

**Published:** 2023-03

**Authors:** TK Amin, I Patel, AH Jangad, H Shah, RP Vyas, NV Patel, DR Modi

**Affiliations:** Department of Orthopaedics, Smt. NHL Municipal Medical College, Ahmedabad, India

**Keywords:** intra-articular fracture, proximal tibia, Schatzker classification, plating, Knee Society Score inclusion

## Abstract

**Introduction:**

Proximal tibial plateau fractures are one of the major problems in orthopaedic surgery and are associated with high complication rates. Intra-articular proximal tibia plateau fractures represent approximately 1% of fractures in adults. Various modalities of proximal tibial plateau fracture management have been considered, ranging from simple external fixators in impending compartment syndrome to periarticular proximal tibia plates and inter-locking nails with poller screws. Purpose of this study is to determine clinical outcomes of proximal tibial plateau fractures treated with plate.

**Materials and methods:**

We did this study of proximal tibial plateau fracture according to Schatzker’s classification treated with proximal tibial periarticular plates in 53 patients prospectively admitted at the author’s institute from June 2018 to May 2020 with follow-up period of 6 months.

**Results:**

In our study, the average knee score was 89.30 (ranging from 79 to 93) and functional knee score was 97.92 (ranging from 75 to 100). Fifty-one (51) patients (96.23%) showed excellent results and 2 patients (3.77%) showed good results according to Knee Society Score, which suggest that internal fixation of proximal tibia plateau fracture with plating provides better results. Out of 53 patients, 9 patients had post-operative complications. Average radiological union was seen at 14 weeks.

**Conclusion:**

Locking compression plate in proximal tibia plateau fractures act as a good biological fixation provide stable fixation, articular reduction and limb alignment even in difficult fracture situations. Fixation of proximal tibia plateau fractures with plate gives excellent to good knee society score, with satisfactory functional and radiological outcome.

## Introduction

Proximal tibia plateau fractures are serious injuries and present treatment challenges. Intra-articular proximal tibia plateau fractures represent approximately 1% of fractures in adults^1^. These fractures include medial and lateral split plateau fracture, bicondylar injuries with significant articular depression; multiple, displaced condylar fracture lines; meta-diaphyseal fracture extension and comminuted injuries^1,2,3^.

They occur from indirect coronal or direct axial compressive forces. These fractures include the medial, lateral or both plateaus with many degrees of articular depression and displacements. Each fracture type has its own characteristic morphology and response to the treatment. It is essential to determine the force of injury since high energy trauma is associated with considerable soft tissue and neurovascular damage. Apart from tibial plateau fractures, meniscal tears and ligament injuries should also be assessed.

Proximal tibial plateau fractures are difficult to treat. The goals of operative treatment of these fractures include anatomic reduction for the restoration of articular congruity and alignment, and stable fixation to allow early mobilisation.

The management of high-energy proximal tibial plateau fracture requires the surgeon to take good care of the soft tissue envelope as the proximal tibia surface is covered only with skin and subcutaneous tissue. Various modalities of complex proximal tibial plateau fracture management have been considered, ranging from simple external fixators in impending compartment syndrome to periarticular proximal tibia plates and inter-locking nails with poller screws^1,2,3^. Here we discuss the clinical outcomes of proximal tibial plateau fractures after surgical management and fixation with plate.

The purpose of this study was to evaluate the post-operative functional and radiological outcome of 53 patients with proximal tibial plateau fractures (Schatzker type I to type VI) treated with plating^4^.

## Materials and Methods

We did this study of proximal tibial plateau fracture treated with proximal tibial periarticular plates in 53 patients prospectively admitted at the author’s institute from June 2018 to May 2020 with follow-up period of 6 months.

Patients with proximal tibial plateau fractures according to Schatzker’s classification^4^ with age above 18 years and Closed and Open type I and II according to Gustilo-Anderson Classification^5^ were included in our study. Patients less than 18 years of age with Open grade III Fracture according to Gustilo-Anderson Classification, pathological fracture and patients requiring vascular repair were excluded from the study.

Besides pre-operative radiographs, CT-Scan with 3D reconstruction of injured knee was carried out to assess the size, location and extension of articular fragment. Temporary stabilisation of fracture with above knee splint was carried out and limb elevation was given. In Gustilo-Anderson type II open fractures temporary external fixator was carried out for soft tissue resuscitation and in fracture with compartment syndrome fasciotomy was done before definitive fracture reconstruction. If the fracture was closed, then patient was posted for surgery after anaesthetic fitness.

Surgeries were performed under spinal anaesthesia on a standard radiolucent or fracture table. Operative limb was painted and draped as per the standard protocol. Tourniquet was inflated when required.

The aim was to achieve anatomical reduction of articular fragment. Sequence of fixation in most of the cases was by indirect reduction and temporary fixation of the articular fracture then alignment of the articular fracture to the diaphysis and fixation with the plate.

**Fig. 1:**
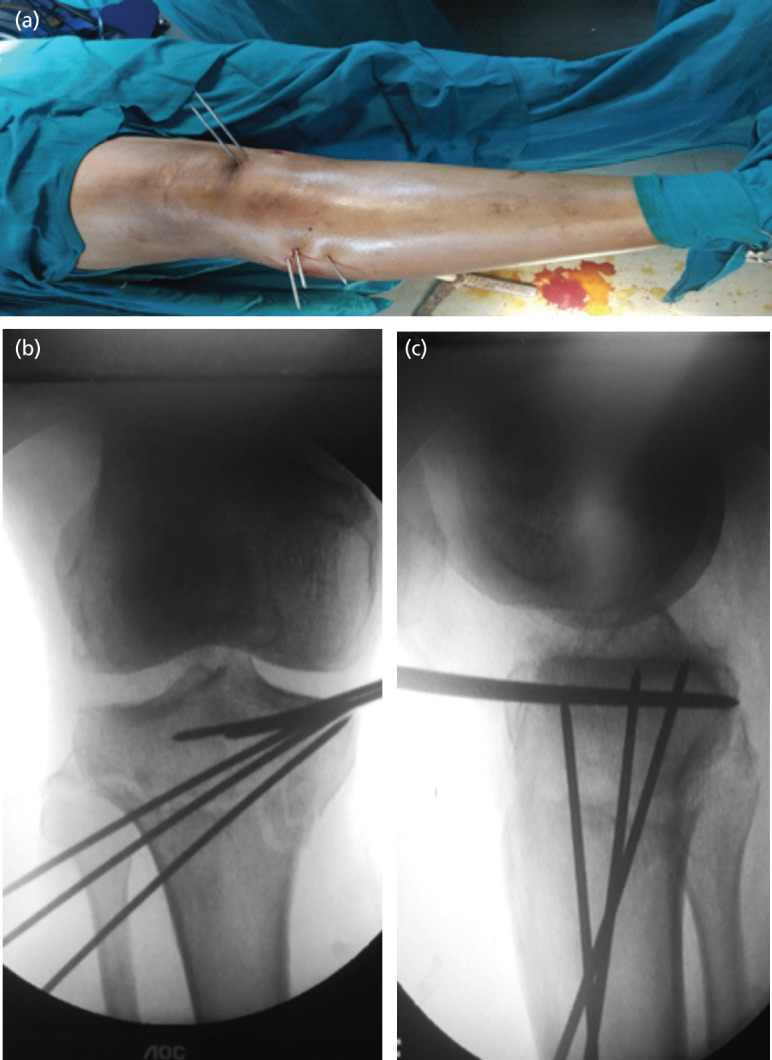
Reduction achieved with traction manipulation and held with K-wire. (a) Clinical intra-operative image. (b and c) Intra-operative C-arm image.

Close reduction was achieved using principle of ligamentotaxis and confirmed under image intensifier, the fracture fragments were held temporarily using Kirschner-wires (K-wire). If fracture reduction was not achieved by closed method, then fracture reduction was tried with joystick method using K-wire or Steinmann pin (Fig. 1). If reduction was not achieved, then open reduction was done. Once the articular reduction was achieved, meta-diaphyseal component was aligned with the articular part and fracture fragments were held in position using K-wire, Steinmann pins or clamps (Fig. 1). Depending upon fracture pattern either lateral, medial, posterior plate alone or in combination were used.

**Fig. 2:**
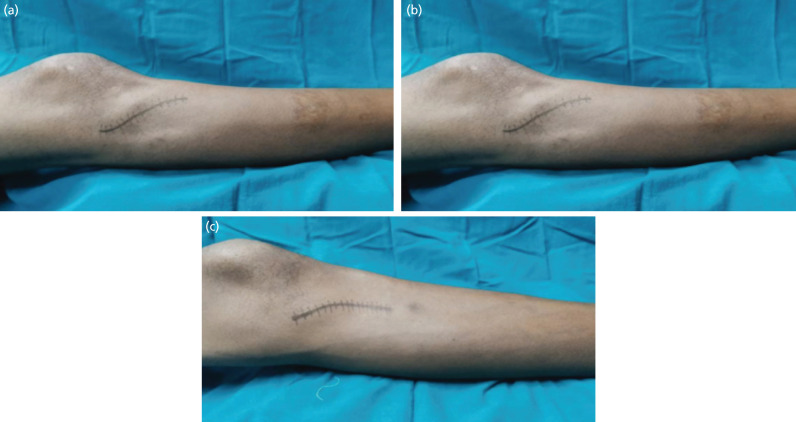
(a) Lateral incision line. (b) Posteromedial incision line.

For lateral plating anterolateral approach was used, approximately 3 to 5cm skin incision^6^ (Fig. 2a) proximal to the joint line in lazy ‘S’ shaped incision was made lateral to the lateral border of the patella tendon. The incision was curved anteriorly over Gerdy’s tubercle and then extended distally, staying about 1cm lateral to the anterior border of the tibia. For posteromedial or medial plating, posteromedial approach was used, in which a 6cm longitudinal skin incision^7^ (Fig. 2b) overlying the posteromedial border of the proximal tibia was made and subcutaneous tunnel was prepared. The exact length of the incision was made depending on the size of the implant used. The appropriate length of the plate was determined by placing a plate along the lateral or medial aspect of the leg and adjusting it so that the proximal end of the plate was just below the joint line and the distal end extended at least three screw holes beyond the distal limit of the tibial fracture under image intensifier. The plate was then slid subcutaneously across the fracture site to reach the distal fragment. Primary plate position was maintained using two K-wires, first in the proximal fragment and second in the distal fragment under the guidance of an image intensifier. After confirming the position of the plate, two simple non-locking screws were used; one in the proximal fragment and another in the distal fragment. The plate was then secured to the condyle with appropriate locking screws. Normal saline wash given, and the wound was closed in layers.

After taking post-operative radiographs removable knee brace with limb elevation was given to the operated limb. Intravenous antibiotics were given for three to five days, and oral antibiotic given till suture removal. Static quadriceps exercises and ankle pump exercises were started on the second post-operative day. The patients were allowed intermittent knee mobilisation once the pain subsided. Sutures were removed on 13-16 days post-operatively. Partial weight bearing was allowed from 4-8 weeks depending upon the fracture configuration and correlation with the radiograph. Full weight bearing was deferred until evidence of union was seen on radiographs (usually by 8-12 weeks).

The course of healing was documented radiologically (AP and lateral) and clinically. Complete healing was defined when radiologically complete bone regeneration was seen at the fracture site and when the patient was pain-free on full weight bearing of the injured limb. Delayed healing was defined as inadequate consolidation at six months after the operation.

Assessment and analysis of any complications were observed and the necessary revision operations with regard to the cause were carried out. Assessment of functional outcome was done using Knee Society Score^8,9^.

## Results

Following observations were made after analysis of patient’s data, intra-operative data, radiological outcome, functional outcome, and results were calculated using the Knee society score. The average age was 40.5 years (ranging from 19 to 85 years), out of which 39 (74%) were male and 14 (26%) were female. This suggests that males were more affected.

In our study, 42 patients (79.24%) were injured due to road traffic accident (RTA), 4 patients (7.55%) due to falling from height and 7 patients (13.21%) due to direct high velocity injury on the leg. This suggests that road traffic accident is the major mode of injury.

According to Schatzker classification, 13 patients (24.5%) had Schatzker type – II fracture, and 13 patients (24.5%) had Schatzker type IV fractures. Thus type-II and type IV-were more common, out of which 48 patients (90.77%) had closed fractures, 4 patients (7.76%) had open grade-I fracture and 1 patient (1.47%) had open grade-II fracture.

The average interval between injury and operation was five days. This delay was mainly due to time required for reduction of soft tissue edema in order to decrease the skin complication and to prevent post-operative compartment syndrome. In six patients definitive fixation was done after five days due to injury related complications in which External fixator was done. Out of 6 patients (11%), 4 patients (7.6%) had open fractures, 1 patient (1.7%) had associated knee dislocation and 1 patient (1.7%) had compartment syndrome which was released by fasciotomy.

Lateral plating was the most commonly used procedure for Schatzker type I, II, III, V and VI. For Schatzker type IV, medial plating was the most widely used technique for the fracture fixation. Unilateral plate was used in fracture having Schatzker type I, II, and III, while dual plating was used in fracture having Schatzker type IV, V, and VI (Table I). The average duration of surgery was 100 minutes.

**Table I:** Surgical procedure according to Schatzker classification.

Procedure	Type I	Type II	Type III	Type IV	Type V	Type VI	Total
Medial / Postero-medial Plating	0	0	0	7	1	0	8
Lateral Plating	9	13	2	2	4	6	36
Dual/Triple plating	0	0	0	4	3	2	9
Total	9	13	2	13	8	8	53

Average time at which partial weight bearing was allowed was eight weeks. Most of the patients were advised partial weight bearing in the form of walker walking between 4 to 8 weeks. One patient was advised partial weight bearing after 3.5 months due to calcaneum fracture in the opposite limb.

Average time at which full weight bearing was allowed was 11 weeks and 4 days. Most of the patients were advised full weight bearing walking with the walker between 8 to 12 weeks. One patient was advised full weight bearing after 4 months due to calcaneum fracture in the opposite limb.

**Fig. 3:**
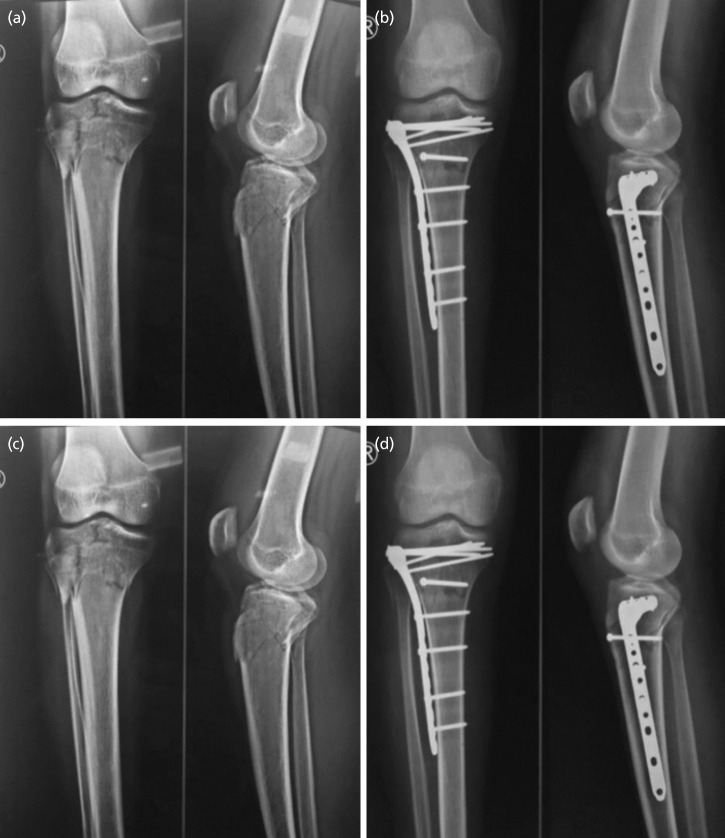
Follow-up series of radiographs. (a) Pre-operative. (b) Immediate post-operative. (c) 2 months post-operative. (d) 6 months post-operative.

Average radiological union was seen at 14 weeks (Table II) (Fig. 3). In this study all the fractures united. The earliest union was seen at 10 weeks and in one patient union was seen at 20 weeks.

**Table II:** Average radiological union time.

Weeks of follow-up	Number of Patients
Less than 13 weeks	19
13 to 15 weeks	17
15 to 17 weeks	15
17 to 20 weeks	2

All the patients in our study had more than 90° of knee flexion. Eleven (11) patients (20.75%) had 125° or more of knee flexion, 26 patients (49.05%) had knee flexion of 115° - 125°, 3 patients (5.66%) had extensor lag of 10°. No patient had joint stiffness and every patient had functional range of movement of knee from near full extension to about 90° of flexion.

In our study, 6 patients (11.32%) had a post-operative infection within two weeks of surgery. These patients were treated with dressings and short course of antibiotics. The infection was controlled by this approach. In 1 patient (1.88%) there was post-operative infection at nearly one year that required removal of the implant.

In our study, 1 patient (1.88%) had a broken plate at 5 months due to trauma. One patient (1.88%) had implant failure at 6 months, but the fracture was united in malposition in both the patients so, it required implant removal, correction of deformity, bone-grafting and re-plating.

In our study, 2 patients (3.77%) had malunion. In which 1 patient (1.88%) had varus deformity of 9° but that deformity did not alter the patient’s activity of daily living. The average knee score (Table III) is 89.30 (Ranging from 79 to 93) and functional knee score is 97.92 (Ranging from 75 to 100). Fifty-one (51) patients (96.23%) showed excellent results.

**Table III:** Knee Society Score.

Knee society score	No. of patients Knee score	Percentage
Excellent (80-100)	51	96.23%
Good (70-79)	2	3.77%
Fair (60-69)	0	-
Poor (<60)	0	-
Total	53	100%
	**Functional score**	
Excellent (80-100)	51	96.23%
Good (70-79)	2	3.77%
Fair (60-69)	0	-
Poor (<60)	0	-
Total	53	100%

## Discussion

Tibial plateau fractures are one of the commonest intra-articular fractures that usually occur as a result of road traffic accident, fall from height, violence etc. For many years, treating proximal tibial fractures has been the subject of much controversy regarding both the indications for surgical intervention and the specific type of intervention to be employed. Especially in intra-articular fractures, inadequate treatment may result in joint instability and deformity coupled with a restricted range of motion^10,11^. Open reduction and rigid internal fixation, according to the principles of Association for Osteosynthesis/Association for the Study of Internal Fixation (AO/ASIF), has been the treatment of choice for decades. This treatment modality has yielded satisfactory short- and long-term results in many series.

Recently, locking plates, or internal fixators, have been designed to allow for less plate to bone contact without compromising stability. The screw holes are modified to allow the screw to “lock” into the plate, thus converting a plate/screw construction into a fixed-angle device with multiple points of fixation^12^. This design allows for minimal vascular damage to the periosteum. Moreover, locking plates can be particularly effective in treating osteoporotic bones^13^.

In our study, we have used Schatzker classification for the proximal tibial plateau fractures type I to VI with the incidence of type-I 9 (16.98%), type-II 13(24.53%), type –III 2 (3.78%), type –IV 13(24.53%), type – V 8 (15.09%) and type –VI 8(15.09%). In Girish H V and co-workers’ study^14^, Schatzker type I and II dominated the total fractures making 50%, with type V and VI having 18.8% and 12.5% involvement, respectively. Similarly, Rademakers *et al*^15^ reported that 64% of patients sustained a lateral condyle fracture (Schatzker type I and II). In MRI analysis of 103 patients, Gardner *et al*^16^ reported that the most frequent fracture pattern was a lateral plateau split-depression (Schatzker type II).

Barei *et al*^17^ showed that the average time interval from injury to definitive surgical treatment was nine days. Manidakis *et al*^18^ showed that the average time interval from injury to definitive surgical treatment was three days. In our study, the average duration from injury to operation was five days. This means that a number of patients had to stay longer before the definitive procedure for (i) general condition stabilisation (ii) oedema to resolve (iii) treatment of compartment syndromes or (iv) open wounds to heal.

In our study of proximal tibia plateau fracture, mean union time was 14 weeks which is comparable to Manidakis *et al*^18^ which showed that average time of union was 13 weeks and Jain *et al*^19^ which showed a series of 34 cases having mean union time of 17.6 weeks.

The most frequently used approach was lateral approach with incidence of 67.92% and the most frequently used implant was lateral anatomical plate (proximal tibial locking plate) which was used in 62.26% of the patients with or without cannulated cancellous screw. In the series by, Pasa *et al*^20^ out of 114 patients with proximal tibial fractures he fixed the fracture with cannulated cancellous screw and washer in 25, and a buttress plate in 27 patients. In the series by Vasanad *et al*^14^ showed that 46.8% of the patients needed ORIF with buttress plate along with cannulated cancellous screw.

In our study, we achieved 96.23% excellent result and 3.77% good result with our standard surgical care using periarticular proximal tibia plating and allowing mobilisation of the knee. Vasanad *et al*^14^ had 44% excellent result and 44% good results (overall 88% acceptable results) (Table IV).

**Table IV:** Comparison of results with different studies.

Authors	Result
Schatzker *et al*^4^	86%
Rademakers *et al*^15^	94%
Manidakis *et al*^18^	69%
Vasanad *et al*^14^	88%
Our study	96%

Kugelman *et al*^21^ showed in their study that out of 279 tibial plateau fractures 10 patients (3.6%) sustained a deep infection. Six patients (2.2%) developed a superficial infection. One patient (0.4%) presented with early implant failure. Two patients (0.7%) developed a fracture non-union. Eight patients (2.9%) developed a venous thromboembolism. Seventeen patients (6.2%) went on to re-operation for symptomatic implant removal. Nine patients (3.3%) underwent a lysis of adhesions procedure. Vasanad *et al*^14^ also showed knee stiffness in three patients, mal-union in two patients, infection and wound dehiscence in three patients, extensor lag in one patient and loss of reduction in one patient. Our study also showed superficial infection in 6 (11.32%) patients which were controlled by antibiotics and dressing. Removal of implants was needed in 2 (3.77%) patients in which 1 (1.88%) patient had delayed infection at one year and one patient (1.88%) had implant failure at 6 months, but fracture had united in mal-position. This patient required implant removal, correction of deformity, bone-grafting and re-fixation with medial and lateral plate. One (1.88%) patient had a varus deformity of 9° but, that deformity did not alter the activities of daily living.

In our study, 97% excellent results as evaluated by Knee Society Score and all the patients had the functional range of movement and low complication rate.

## Conclusion

Locking compression plate in proximal tibia plateau fractures act as a good biological fixation provide stable fixation, articular reduction and limb alignment even in difficult fracture situations. Fixation of proximal tibia plateau fractures with plate gives excellent to good knee society score, faster union time with satisfactory functional and radiological outcome.

## References

[1] Marsh JL, Karam MD, Court-Brown CM, Heckman JD, McQueen MM, Ricci WM, Tornetta P, McKee MD (2015). Rockwood and Green’s Fractures in adults..

[2] Chapman MW, Szabo RM, Marder R, Vince KG, Mann RA, Lane JM (2001). Chapman's Orthopaedic Surgery..

[3] Lowe JA, Tejwani N, Yoo B, Wolinsky P (2011). Surgical techniques for complex proximal tibial fractures.. J Bone Joint Surg Am..

[4] Schatzker J, McBroom R, Bruce D (1979). The tibial plateau fracture. The Toronto experience 1968--1975.. Clin Orthop Relat Res..

[5] Gustilo RB, Merkow RL, Templeman D (1990). The management of open fractures.. J Bone Joint Surg Am..

[6] Kaplan EB (1957). Surgical approach to the lateral (peroneal) side of the knee joint.. Surg Gynecol Obstet..

[7] Hughston JC (1973). A surgical approach to the medial and posterior ligaments of the knee.. Clin Orthop Relat Res..

[8] Insall JN, Dorr LD, Scott RD, Scott WN (1989). Rationale of the Knee Society clinical rating system.. Clin Orthop Relat Res..

[9] Asif S, Choon DS (2005). Midterm results of cemented Press Fit Condylar Sigma total knee arthroplasty system.. J Orthop Surg (Hong Kong)..

[10] Yu B, Han K, Ma H, Zhang C, Su J, Zhao J (2009). Treatment of tibial plateau fractures with high strength injectable calcium sulphate.. Int Orthop..

[11] Stevens DG, Beharry R, McKee MD, Waddell JP, Schemitsch EH (2001). The long-term functional outcome of operatively treated tibial plateau fractures.. J Orthop Trauma..

[12] Greiwe RM, Archdeacon MT (2007). Locking plate technology: current concepts.. J Knee Surg..

[13] Fulkerson E, Egol KA, Kubiak EN, Liporace F, Kummer FJ, Koval KJ (2006). Fixation of diaphyseal fractures with a segmental defect: a biomechanical comparison of locked and conventional plating techniques.. J Trauma..

[14] Vasanad GH, Antin SM, Akkimaradi RC, Policepatil P, Naikawadi G (2013). "Surgical management of tibial plateau fractures - a clinical study".. J Clin Diagn Res..

[15] Rademakers MV, Kerkhoffs GM, Sierevelt IN, Raaymakers EL, Marti RK (2007). Operative treatment of 109 tibial plateau fractures: five- to 27-year follow-up results.. J Orthop Trauma..

[16] Gardner MJ, Yacoubian S, Geller D, Suk M, Mintz D, Potter H (2005). The incidence of soft tissue injury in operative tibial plateau fractures: a magnetic resonance imaging analysis of 103 patients.. J Orthop Trauma..

[17] Barei DP, Nork SE, Mills WJ, Coles CP, Henley MB, Benirschke SK (2006). Functional outcomes of severe bicondylar tibial plateau fractures treated with dual incisions and medial and lateral plates.. J Bone Joint Surg Am..

[18] Manidakis N, Dosani A, Dimitriou R, Stengel D, Matthews S, Giannoudis P (2010). Tibial plateau fractures: functional outcome and incidence of osteoarthritis in 125 cases.. Int Orthop..

[19] Jain D, Selhi H, Mahindra P, Kohli S, Yamin M (2012). Results of proximal tibial fractures managed with periarticular locking plates: A series of 34 cases.. Indian J Res Rep Med Sci..

[20] Pasa L, Kelbl M, Suchomel R, Procházka V, Filipínský J (2007). Výsledky lécby niktrokloubních zlomenin proximální tibie v UN Brno v letech 1997 az 1999: hodnocení po 5-7 letech od terapie [Treatment of intra-articular proximal tibial evaluation of two- to seven-year follow-up].. Acta Chir Orthop Traumatol Cech..

[21] Kugelman D, Qatu A, Haglin J, Leucht P, Konda S, Egol K (2017). Complications and unplanned outcomes following operative treatment of tibial plateau fractures.. Injury..

